# Reduction in acute migraine-specific and non-specific medication use in patients treated with erenumab: post-hoc analyses of episodic and chronic migraine clinical trials

**DOI:** 10.1186/s10194-021-01292-w

**Published:** 2021-07-23

**Authors:** Stewart J Tepper, Messoud Ashina, Uwe Reuter, Yngve Hallström, Gregor Broessner, Jo H Bonner, Hernan Picard, Sunfa Cheng, Denise E Chou, Feng Zhang, Jan Klatt, Daniel D Mikol

**Affiliations:** 1 Geisel School of Medicine at Dartmouth, Hanover, NH USA; 2 Danish Headache Center, Department of Neurology, Faculty of Health and Medical Sciences, Rigshospitalet Glostrup, University of Copenhagen, Copenhagen, Denmark; 3 Department of Neurology, Charité Universitätsmedizin Berlin, Berlin, Germany; 4 Neuro Center, St Görans Hospital, Stockholm, Sweden; 5 Department of Neurology, Headache Outpatient Clinic, Medical University of Innsbruck, Innsbruck, Austria; 6 Mercy Clinic Neurology and Headache Centre, Saint Louis, MO USA; 7 Amgen Inc, Thousand Oaks, CA USA; 8 Novartis Pharma AG, CH-4002 Basel, Switzerland

**Keywords:** CGRP receptor, Chronic migraine, Episodic migraine, Erenumab, Migraine-specific

## Abstract

**Background:**

In patients with migraine, overuse of acute medication, including migraine-specific medication (MSM) such as triptans and ergots, can lead to adverse health outcomes, including development of medication overuse headache. Here, we examined the effect of erenumab on reducing acute medication use, in particular MSM, in patients with episodic migraine (EM) and chronic migraine (CM).

**Methods:**

The current post-hoc analyses were based on data from the double-blind treatment phase (DBTP) of two erenumab studies, a pivotal EM (*N* = 955) and a pivotal CM (*N* = 667) trial, and their respective extensions. Patients were administered subcutaneous placebo or erenumab (70 or 140 mg) once monthly. Daily acute headache medication use (including MSM and non-MSM) was recorded using an electronic diary during a 4-week pretreatment baseline period until the end of the treatment period. Outcome measures included change in monthly acute headache medication days (HMD) in acute headache medication users at baseline, and changes in monthly MSM days (MSMD) in MSM users at baseline and non-MSMD in non-MSM users at baseline.

**Results:**

In total, 60 and 78 % of patients (all acute headache medication users) with EM and CM used MSM at baseline, respectively. For acute headache medication users, the change in mean monthly acute HMD over Months 4, 5 and 6 compared with the pre-DBTP was 1.5, 2.5, and 3.0 for placebo, erenumab 70 mg and 140 mg, respectively for the EM study. The respective change in monthly MSMD in MSM users was 0.5, 2.1 and 2.8, and in monthly non-MSMD in non-MSM users was 2.3, 2.6, and 2.7. In the acute headache medication users at baseline, the change in monthly acute HMD at Month 3 compared with pre-DBTP was 3.4, 5.5, and 6.5 for placebo, erenumab 70 mg and 140 mg, respectively for the CM study. The respective change in monthly MSMD in MSM users was 2.1, 4.5, and 5.4, and in monthly non-MSMD in non-MSM users was 5.9, 6.4, and 6.6. Reductions in MSMD versus placebo were sustained in the extension periods of both studies. Erenumab was also associated with a higher proportion of MSM users achieving ≥ 50 %, ≥ 75 and 100 % reduction from baseline in monthly MSMD versus placebo in both EM and CM.

**Conclusions:**

In both EM and CM, treatment with erenumab is associated with a significant and sustained reduction in the use of acute headache medication, in particular MSM.

**Trial registrations:**

NCT02456740; NCT02066415; NCT02174861.

## Background

Many patients with migraine rely on the use of acute medication, both migraine-specific and non-specific, for the symptomatic relief of a migraine attack. In the American Migraine Prevalence and Prevention (AMPP) survey (*N* = 162,576), 98 % of respondents reported using acute medications for the treatment of a migraine attack, with a respective 49 %, 20 %, and 29 % using over-the-counter (OTC), prescription, or both prescription and OTC medications [1].

Excessive use of acute medication in the treatment of migraine is of clinical concern. The International Classification of Headache Disorders 3rd edition (ICHD-3) defines medication overuse as triptan, opioid, barbiturate, ergot alkaloid medication, or combination analgesic use on at least 10 days per month, or a non-steroidal anti-inflammatory drug (NSAID) or simple analgesic on at least 15 days per month [2]. Results from the Migraine in America Symptoms and Treatment (MAST) study reported that 15 % of a broad population of 13,649 adults with migraine surveyed overused acute medications according to the ICHD-3 criteria, with the proportion increasing to 41 % in those with severe disease (≥ 15 monthly migraine days [MMD]) [3]. The overuse of any analgesics or migraine-specific medication (MSM), excluding gepants, which are not thought to cause migraine overuse headache (MOH) with frequent use, can lead to MOH as well as serious medical health consequences, and, therefore, a reduction in acute medication is an important goal in migraine management for both prevention and treatment of MOH [2–4]. In fact, according to the International Headache Society, the most common cause of symptoms associated with chronic migraine (CM) is medication overuse [2]. A reduction in acute medication use has additional potential patient benefits in reducing the overall medication burden and risk of health adverse events associated with both migraine-specific and non-specific medications [5], resulting in potential reductions in healthcare resource use [6–8].

Subcutaneous erenumab, a fully human IgG2 monoclonal antibody (mAb), is a highly potent and selective antagonist of the canonical CGRP receptor [9, 10]. Erenumab has previously been shown to reduce MMD and increase the likelihood of achieving a clinically meaningful response at all monthly assessment points tested in placebo-controlled trials, including pivotal trials in episodic migraine (EM) [11] and CM [12]. Long-term data demonstrate that erenumab remains well tolerated and that its efficacy, including reductions in acute medication use, is sustained [13–15]. Furthermore, it has been shown that more than half of those treated with erenumab convert from CM to EM and from acute migraine medication overuse to no overuse status [16].

Reductions in the use of acute MSM (specifically, triptans, ergots) associated with erenumab have been published for the overall populations of the EM [11] and CM [12] studies. The protocols for both the EM and CM studies specified that patients could use any type of acute headache medication, albeit with limitations on the use of opioids and butalbital-containing medications, if ongoing at baseline; however, use of new acute headache medication could not be initiated post-baseline. Therefore, according to the protocol, MSM use was not relevant to the subgroup of non-MSM users at baseline. Similarly, non-MSM use was only assessed in patients who took non-MSM at baseline. In this report, we focus on the effects of erenumab versus placebo on monthly MSM treatment days (MSMD) when restricted to the subgroup of MSM users at baseline. In addition, we assess the effects of erenumab versus placebo on two further endpoints: (1) the use of all acute headache medication (which includes MSM and non-MSM treatments, such as paracetamol/acetaminophen, combination analgesics, and NSAIDs) in the acute headache medication users at baseline and (2) use of ***only*** non-migraine-specific acute headache medication (non-MSM, in the subgroup of patients using only non-MSM at baseline).

## Methods

### Study design

Analyses were based on data from two erenumab studies; a pivotal EM and a pivotal CM trial, and their extensions.

The study design, and the results for the primary endpoint and key secondary endpoints of the STRIVE (NCT02456740) study, which enrolled patients with EM, have been published previously [11, 14]. In brief, patients were randomized to placebo, or erenumab 70 mg or 140 mg once monthly (QM) during the 24-week, placebo-controlled, double-blind treatment phase (DBTP) of the study. Patients were then re-randomized (1:1) to erenumab 70 mg or 140 mg in a dose-blinded fashion and entered a further 28-week active treatment phase (ATP). In both the DBTP and ATP portions of the STRIVE study, efficacy was assessed throughout.

The study design, primary and key secondary endpoints of the CM study (NCT02066415) have also been published previously [12, 13]. In brief, patients were randomized to placebo, or erenumab 70 mg or 140 mg QM during the 12-week placebo-controlled DBTP, which was followed by a 52-week open-label treatment phase (OLTP; NCT02174861). Initial participants in the OLTP received 70 mg erenumab QM. However, a subsequent protocol amendment increased the dosage to 140 mg QM. Patients who had completed the Week 28 visit before the protocol amendment received erenumab 70 mg throughout, while patients who enrolled in the OLTP, but had not completed the Week 28 visit before the protocol amendment, received 140 mg erenumab at the next possible visit and thereafter. Patients who enrolled in the OLTP after protocol amendment received erenumab 140 mg throughout. Efficacy was assessed during the entire 12-week DBTP and intermittently at Weeks 1–12, 21–24, 37–40, and 49–52 of the OLTP. An independent ethics committee at all participating sites for each clinical trial approved the study protocols.

### Analysis of monthly acute headache medication, MSM, and non-MSM use

Patients recorded acute headache medication (including MSM and non-MSM) use with a daily electronic diary throughout the 4-week baseline period and subsequent treatment periods. Patients could use any type of acute headache medication (MSM or non-MSM) for the acute treatment of their headaches as long as they were being used at baseline; however, use of new acute headache medication could not be initiated post-baseline. The number of days per month on which MSM and non-MSM were taken was calculated based on daily diary data with missing diary entries handled using the proration method. Patients who had ≥ 1 MSMD at baseline were considered a MSM user. Patients who did not use any acute headache medication at baseline were excluded from this analysis.

The pre-DBTP baseline (i.e., monthly measurement based on data collected in the four weeks prior to the first dose of erenumab or placebo in the DBTP) was used to calculate change from baseline efficacy endpoints in the DBTP and the extension periods. The primary efficacy endpoint in the DBTP was analyzed based on mean over Months 4, 5 and 6 (EM study) [11] and at Month 3 (CM study) [12].

Results for the EM ATP are presented by dose received during the 28-week ATP (‘*all erenumab 70 mg’* and ‘*all erenumab 140 mg’* groups, each of which included patients who received either placebo, erenumab 70 mg or erenumab 140 mg during the 24-week DBTP). At the end of the 24-week DBTP, patients were re-randomized in a dose-blinded fashion to either 70 mg or 140 mg for the remainder of the study.

Given that patients in the CM OLTP were not randomized or blinded to erenumab 70 mg or 140 mg, and that some subjects switched dose from 70 mg to 140 mg at variable time points before Week 28, the results for the CM OLTP are presented by last dose received at Weeks 40 and 52. By Week 40, any patient who had a dose increase to 140 mg, at or before Week 28, had received 140 mg for at least 12 weeks and therefore had reached steady state.

MSMD responder analyses in MSM users at baseline were conducted as follows: the proportion of patients achieving ≥ 50 %, ≥ 75 %, and 100 % reduction in MSMD from pre-DBTP baseline to the end of the DBTPs, and to the end of the associated extension periods, were measured. In addition, the proportion of patients with no response to treatment, defined as no change or increase (worsening) in MSMD, was assessed.

Change from baseline in monthly MSMD in MSM users at baseline and monthly non-MSMD in non-MSM users during the DBTPs was analyzed separately using a linear mixed effects model, including covariates of treatment, visit, treatment by visit interaction, stratification factors (region and migraine preventive medication status for EM; region and medication overuse status for CM), and baseline value. The MSMD responder and non-responder endpoints during the DBTP were analyzed using the Cochran-Mantel-Haenszel (CMH) test stratified by stratification factors. Nominal p-values for comparison between erenumab and placebo during the DBTP were provided without multiplicity adjustment. A descriptive summary is provided for efficacy data in the extension periods.

## Results

### MSM users and non-MSM only users at baseline

Baseline characteristics of the acute headache medication users and subgroups of interest are presented in Table 1. In the EM and CM studies, 60 and 78 % of patients (all acute headache medication users at baseline) utilized MSM at baseline, respectively. A higher proportion of patients in the MSM subgroup had prior preventive treatment compared to the non-MSM subgroup; for both EM and CM, approximately twice as many patients were preventive treatment-naïve in non-MSM only users.

**Table 1:** Baseline characteristics of the overall population and subgroups of interest by dose received during the DBTP

	Acute headache medication users at baseline			Users of migraine-specific medicine at baseline				Non-users of migraine-specific medicine at baseline			
**Episodic migraine**											
	Placebo (*N* = 315)	70 mg (*N* = 305)	140 mg (*N* = 312)		Placebo (*N* = 191)	70 mg (*N* = 179)	140 mg (*N* = 192)		Placebo (*N* = 124)	70 mg (*N* = 126)	140 mg (*N* = 120)
Demographics											
Age, years (range)	41.3 ± 11.2 (18–65)	41.2 ± 11.2 (18–63)	40.4 ± 11.1 (19–65)		43.3 ± 10.9 (18–65)	43.8 ± 11 (18–63)	42 ± 10.7 (19–65)		38.4 ± 11 (18–64)	37.5 ± 10.6 (19–63)	37.9 ± 11.4 (19–62)
Female, n (%)	271 (86.0)	261 (85.6)	266 (85.3)		164 (85.9)	152 (84.9)	169 (88)		107 (86.3)	109 (86.5)	97 (80.8)
Caucasian, n (%)	274 (87.0)	274 (89.8)	288 (92.3)		180 (94.2)	173 (96.6)	184 (95.8)		94 (75.8)	101 (80.2)	104 (86.7)
Baseline characteristics											
Monthly migraine days	8.3 ± 2.5	8.3 ± 2.5	8.3 ± 2.5		8.5 ± 2.5	8.4 ± 2.5	8.5 ± 2.4		7.9 ± 2.5	8.0 ± 2.3	8.1 ± 2.5
Monthly headache days	9.3 ± 2.6	9.0 ± 2.6	9.3 ± 2.5		9.3 ± 2.6	9.0 ± 2.6	9.3 ± 2.5		9.3 ± 2.6	9.0 ± 2.6	9.2 ± 2.6
Monthly migraine-specific medication days	3.5 ± 3.4	3.3 ± 3.4	3.5 ± 3.5		5.7 ± 2.6	5.7 ± 2.5	5.7 ± 2.7		0	0	0
Age at migraine onset	21.2 ± 10.1	21.2 ± 11	20.7 ± 9.9		20.6 ± 10.2	21.5 ± 11.4	21.2 ± 9.8		22.0 ± 10	20.7 ± 10.5	20.0 ± 10.2
Disease duration, years	20.2 ± 12.2	20.1 ± 12.2	19.8 ± 12.3		22.7 ± 11.9	22.4 ± 12.1	20.9 ± 12.2		16.4 ± 11.5	16.8 ± 11.5	18.0 ± 12.3
Acute headache medication use, n (%)											
Any	315 (100)	305 (100)	312 (100)		191 (100)	179 (100)	192 (100)		124 (100.0)	126 (100.0)	120 (100.0)
Migraine-specific	191 (60.6)	179 (58.7)	192 (61.5)		191 (100)	179 (100)	192 (100)		0 (0)	0 (0)	0 (0)
Non-migraine-specific	244 (77.5)	242 (79.3)	256 (82.1)		120 (62.8)	116 (64.8)	136 (70.8)		124 (100.0)	126 (100.0)	120 (100.0)
Migraine-preventive medication use, n (%)											
Naïve	174 (55.2)	164 (53.8)	184 (59.0)		84 (44)	73 (40.8)	87 (45.3)		90 (72.6)	91 (72.2)	97 (80.8)
Previous	131 (41.6)	132 (43.3)	120 (38.5)		100 (52.4)	100 (55.9)	100 (52.1)		31 (25.0)	32 (25.4)	20 (16.7)
History of treatment failure of preventive medication*	127 (40.3)	126 (41.3)	112 (35.9)		98 (54.7)	98 (54.7)	92 (47.9)		28 (22.6)	28 (22.2)	20 (16.7)
History of migraine with aura, n (%)	160 (50.8)	162 (53.1)	157 (50.3)		82 (42.9)	82 (45.8)	84 (43.8)		78 (62.9)	80 (63.5)	73 (60.8)
**Chronic migraine**											
	Placebo (*N* = 282)	70 mg (*N* = 191)	140 mg (*N* = 188)		Placebo (*N* = 225)	70 mg (*N* = 143)	140 mg (*N* = 149)		Placebo (*N* = 57)	70 mg (*N* = 48)	140 mg (*N* = 39)
Demographics											
Age, years (range)	42.2 ± 11.3 (18–66)	41.4 ± 11.3 (18–64)	43.1 ± 11 (18–64)		43.4 ± 11.1 (18–66)	42.5 ± 11 (18–64)	44.4 ± 10.3 (18–64)		37.7 ± 11 (18–64)	38.1 ± 11.7 (18–58)	38.3 ± 12.3 (20–58)
Female, n (%)	223 (79.1)	166 (86.9)	158 (84)		175 (77.8)	126 (88.1)	134 (89.9)		48 (84.2)	40 (83.3)	24 (61.5)
Caucasian, n (%)	264 (93.6)	176 (92.1)	182 (96.8)		215 (95.6)	134 (93.7)	146 (98)		49 (86)	42 (87.5)	36 (92.3)
Baseline characteristics											
Monthly migraine days	18.2 ± 4.7	17.9 ± 4.4	17.7 ± 4.6		18.2 ± 4.6	18.1 ± 4.4	17.7 ± 4.3		18.1 ± 5.3	17.1 ± 4.4	17.5 ± 5.9
Monthly headache days	21.1 ± 3.9	20.5 ± 3.8	20.7 ± 3.8		20.9 ± 3.8	20.6 ± 3.8	20.3 ± 3.7		21.8 ± 4.3	20.3 ± 4	21.9 ± 3.8
Monthly migraine-specific medication days	9.6 ± 7.6	8.8 ± 7.2	9.8 ± 7.0		12.0 ± 6.5	11.7 ± 5.8	12.3 ± 5.5		0	0	0
Age at migraine onset	20.3 ± 10.0	21.1 ± 10.5	21.5 ± 10.7		20.7 ± 10.1	19.9 ± 10.2	21.1 ± 10.8		18.8 ± 9.4	24.7 ± 10.9	23.2 ± 10.4
Disease duration, years	22.4 ± 12.6	20.7 ± 12.8	22.1 ± 11.8		23.1 ± 12.8	23.0 ± 12.9	23.8 ± 11.2		19.4 ± 11.7	13.9 ± 10.2	15.6 ± 11.9
Acute headache medication use, n (%)											
Any	282 (100)	191 (100)	188 (100)		225 (100)	143 (100)	149 (100)		57 (100)	48 (100)	39 (100)
Migraine-specific	225 (79.8)	143 (74.9)	149 (79.3)		225 (100)	143 (100)	149 (100)		0 (0)	0 (0)	0 (0)
Non-migraine-specific	246 (87.2)	167 (87.4)	161 (85.6)		189 (84)	119 (83.2)	122 (81.9)		57 (100)	48 (100)	39 (100)
Migraine-preventive medication use, n (%)											
Naïve	67 (23.8)	53 (27.7)	54 (28.7)		41 (18.2)	31 (21.7)	31 (20.8)		26 (45.6)	22 (45.8)	23 (59.0)
Previous	215 (76.2)	138 (72.3)	134 (71.3)		184 (81.8)	112 (78.3)	118 (79.2)		31 (54.4)	26 (54.2)	16 (41.0)
History of treatment failure of preventive medication^a^	198 (70.2)	127 (66.5)	124 (66)		170 (75.6)	105 (93.8)	111 (94.1)		28 (49.1)	22 (45.8)	13 (33.3)
History of migraine with aura, n (%)	122 (43.3)	81 (42.4)	70 (37.2)		93 (41.3)	58 (40.6)	61 (40.9)		29 (50.9)	23 (47.9)	9 (23.1)

^a^≥1 failed preventive medication

Plus–minus values are means ± SD. Data in the table are for the full analysis set (all patients who underwent randomization)

### Effect of erenumab on acute headache medication, MSM and non-MSM use

**Fig. 1:**
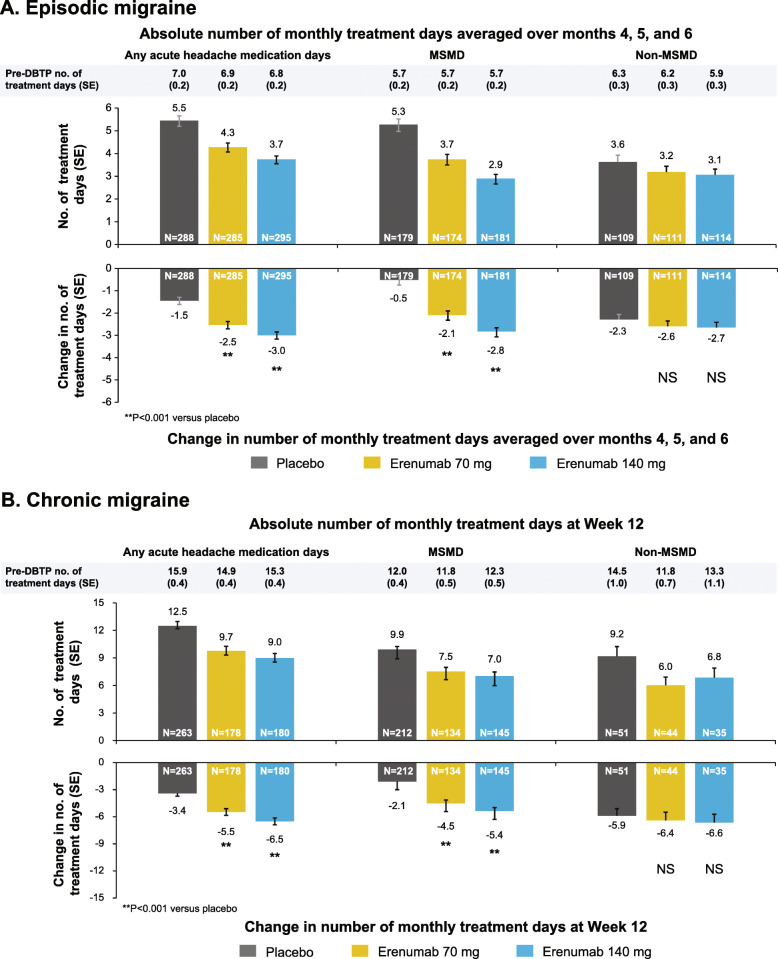
Change from pre-DBTP in monthly days of any acute headache medication, MSM, or non-MSM use. The change from pre-DBTP in monthly days of any acute headache medication, MSM, or non-MSM use at (**A**) mean over Months 4, 5, and 6 (EM) and (**B**) Month 3 (CM) is shown. Baseline values are mean ± SD; change from baseline values are adjusted analysis utilizing a generalized linear mixed model which includes treatment, visit, treatment by visit interaction, stratification factors and baseline value as covariates and assuming a first-order autoregressive covariance structure. DBTP, double-blind treatment phase; MSM, migraine-specific medication; non-MSM, non-migraine-specific medication, NS, non-significant; SD, standard deviation

Figure 1 shows the change from pre-DBTP baseline in the mean number of monthly acute HMD, MSMD (in MSM users at baseline) and non-MSMD (in non-MSM only users at baseline) by DBTP treatment groups in patients with EM during Months 4–6 and CM at Month 3.

### Effect of erenumab on monthly MSMD in patients with EM over time

#### (MSM users at baseline)

**Fig. 2:**
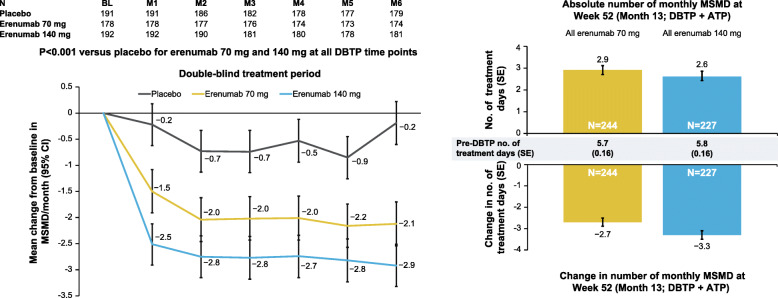
Change in monthly MSMD over DBTP and ATP of the EM study in users of MSM at baseline. Baseline values are not adjusted analysis; DBTP results are presented as adjusted means and 95 % CIs utilizing a generalized linear mixed model which includes treatment, visit, treatment by visit interaction, stratification factors region and prior/current treatment with migraine prophylactic medication, and baseline value as covariates and assuming a first-order autoregressive covariance structure. P-values for pairwise comparisons are nominal p-values without multiplicity adjustment. ATP results are presented as mean ± SE. ‘*All erenumab 70 mg’* in the ATP at Week 52 comprised patients with the following DBTP treatment assignments: placebo (*n* = 83), erenumab 70 mg (*n* = 73), erenumab 140 mg (*n* = 88). ‘*All erenumab 140 mg’* in the ATP comprised patients with the following DBTP treatment assignments: placebo (*n* = 77), erenumab 70 mg (*n* = 77), erenumab 140 mg (*n* = 73). ATP, active treatment phase, CI, confidence interval; DBTP, double-blind treatment phase; EM, episodic migraine; M, Month; MSM, migraine-specific medication; MSMD, migraine-specific medication days; SE, standard error

In the EM study, both doses of erenumab significantly reduced MSMD versus placebo in the subgroup of patients using MSM at baseline (Figs. 1 and 2). For placebo, the pre-DBTP number of MSMD and the mean number of MSMD over Months 4, 5, and 6 (SE) where MSMD is a monthly measure for EM, were 5.7 days (0.2) and 5.3 days (0.3), respectively (change from baseline ‒0.5 [0.2]). The corresponding mean pre-DBTP, over Months 4, 5, and 6, and change from baseline values for erenumab 70 mg were 5.7 (0.2), 3.7 (0.2), and ‒2.0 (0.2), respectively (LSM difference [95 % CI] versus placebo − 1.6 days [− 2.0, − 1.1], p < 0.001). For erenumab 140 mg these values were 5.7 (0.2), 2.9 (0.2), and ‒2.7 (0.2) (LSM difference [95 % CI] versus placebo − 2.3 days [− 2.8, − 1.8], p < 0.001]). A difference for both doses versus placebo was observed at Week 4 and was sustained throughout the DBTP, while a reduction in the number of MSMD was also sustained throughout the DBTP and the ATP (Fig. 2).

### Effect of erenumab on monthly MSMD in patients with CM over time

#### (MSM users at baseline)

**Fig. 3:**
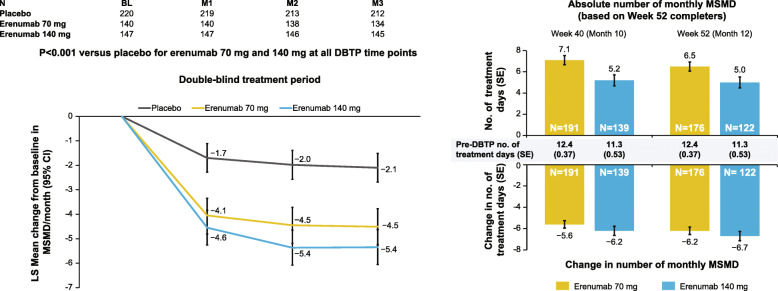
Change in monthly MSMD over DBTP and OLTP of the CM study in users of MSM at baseline. DBTP results are presented as adjusted means and 95 % CIs utilizing a generalized linear mixed model which includes treatment, visit, treatment by visit interaction, stratification factors region and medication overuse status, and baseline value as covariates and assuming a first-order autoregressive covariance structure. P-values for pairwise comparisons are nominal p-values without multiplicity adjustment. OLTP results are presented as mean ± SE. For patients switching from 70 mg to 140 mg between Weeks 4 and 28 of the OLTP, by Week 40 patients would have been on 140 mg for at least 12 weeks and would have therefore achieved steady state. Consequently, by Week 52, patients would have been on 140 mg for at least 24 weeks. DBTP, double-blind treatment phase; CI, confidence interval; CM, chronic migraine; M, month; MSM, migraine-specific medication; MSMD, migraine-specific medication days; OLTP, open-label treatment phase; SE, standard error

In the CM study, the pre-DBTP number of monthly MSMD and the number of monthly MSMD at Month 3 (SE) in the placebo group were 12.0 days (0.4) and 9.9 days (0.5), respectively (change from baseline ‒2.1 [0.3]). The corresponding pre-DBTP, Month 3, and change from baseline values for erenumab 70 mg were 11.8 (0.5), 7.5 (0.5), and ‒4.3 (0.5) (LSM difference [95 % CI] versus placebo − 2.4 [–3.4, − 1.5]). For erenumab 140 mg, these values were 12.3 (0.5), 7.0 (0.5), and − 5.4 (0.4) (LSM difference [95 % CI] versus placebo − 3.3 days [–4.2, − 2.3]). Figure 3 shows the early and pronounced treatment difference in MSM users at baseline, which was sustained throughout the DBTP and the OLTP.

### MSM responder analysis in MSM users at baseline

**Fig. 4:**
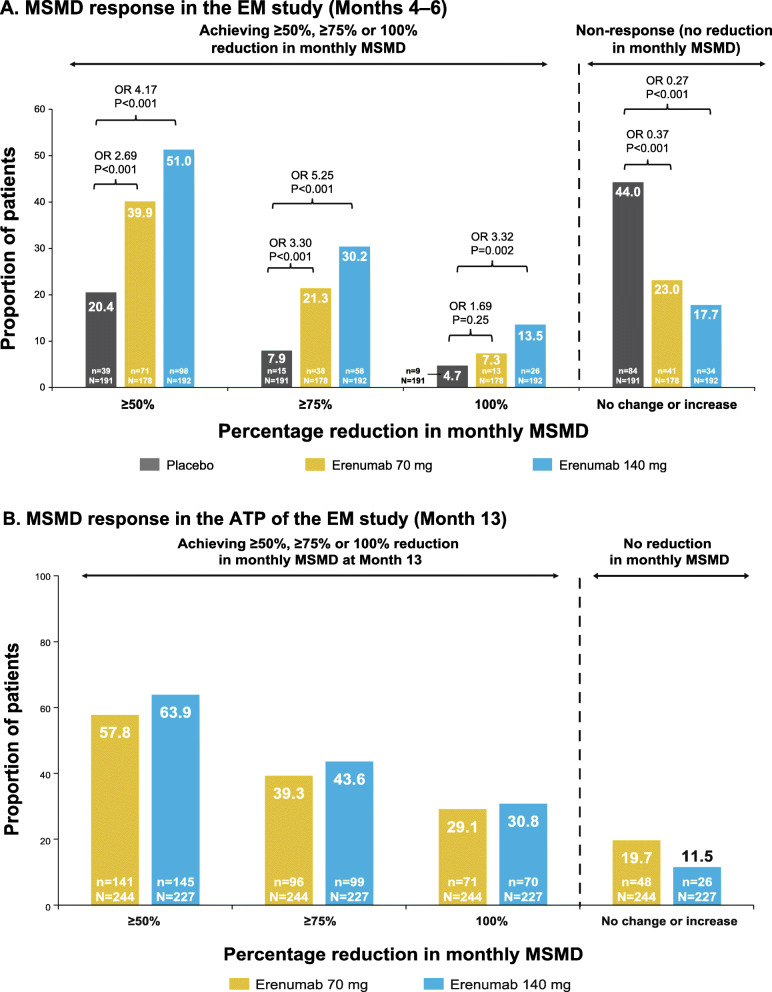
Proportion of patients achieving ≥ 50, ≥75 and 100 % reduction from baseline in monthly MSMD in EM study through (**A**) DBTP and (**B**) ATP. The common odds ratios and p-values are obtained from a CMH test, stratified by stratification factors region and prior/current treatment with migraine prophylactic medication. P-values for pairwise comparisons are nominal p-values obtained from the CMH test using data including placebo and corresponding erenumab dose group only. ATP, active treatment phase; CMH, Cochran-Mantel-Haenszel, EM, episodic migraine; MSMD, migraine-specific medication days; OR, odds ratio

**Fig. 5:**
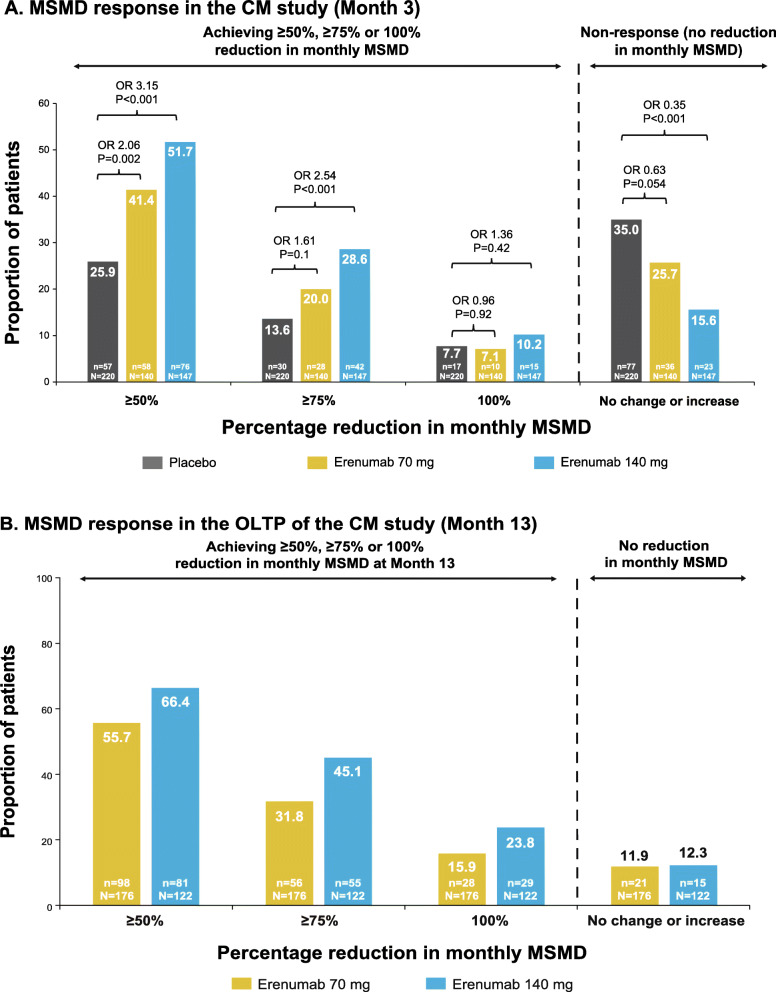
Proportion of patients achieving ≥ 50, ≥75 and 100 % reduction from baseline in monthly MSMD in CM study through (**A**) DBTP and (**B**) OLTP. The common odds ratios and p-values are obtained from a CMH test, stratified by stratification factors region and medication overuse status. P-values for pairwise comparisons are nominal p-values obtained from the CMH test using data including placebo and corresponding erenumab dose group only. CM, chronic migraine; CMH, Cochran-Mantel-Haenszel, MSMD, migraine-specific medication days; OLTP, open-label treatment phase; OR, odds ratio

In both EM and CM, erenumab 70 mg and erenumab 140 mg were associated with a higher proportion of patients achieving a ≥ 50 %, ≥ 75 %, and 100 % reduction from baseline in monthly MSMD compared with placebo. Additionally, numerically higher proportions of patients achieving these reductions were seen for erenumab 140 mg compared with erenumab 70 mg; this was also true for the ATP of the EM study and the OLTP of the CM study (Figs. 4 and 5).

## Discussion

These analyses demonstrate that treatment with erenumab was associated with significant reductions in monthly acute HMD (any acute medication days and MSMD in the DBTPs of the pivotal EM and CM clinical studies). Corresponding numerical reductions were also observed for non-MSMD in non-MSM only users at baseline. Further analyses demonstrated that the reductions in MSMD were maintained in the extension phases of the EM and CM studies.

### Effect of erenumab on MSMD in the acute headache medication users versus MSM users at baseline

Reductions in MSMD associated with erenumab, using the entire study populations of the EM and CM studies, have been reported previously [11, 12]. However, given that not all patients used MSM at baseline, we performed a more relevant analysis using the subgroup of MSM users at baseline and the results reflect the expected greater effect in this subgroup. In the acute headache medication users of the EM study, the mean number of monthly MSMD pre-DBTP and over Months 4, 5 and 6 (where MSMD is a monthly measure for EM), respectively, for placebo were 3.4 days and 3.3 days, for erenumab 70 mg were 3.2 and 2.3, and for erenumab 140 mg were 3.4 and 1.8 (relative reductions versus placebo over Months 4, 5, and 6 of − 0.9 and − 1.4 for the 70 mg and 140 mg doses, respectively). In the current analysis, the corresponding relative reductions in the subgroup of MSM users at baseline were − 1.6 days and − 2.3 days.

In the acute headache medication users of the CM study, the mean number of monthly MSMD pre-DBTP and at Month 3, respectively, for placebo were 9.5 days and 7.9 days, for erenumab 70 mg were 8.8 and 5.7, and for erenumab 140 mg were 9.7 and 5.6 (relative reductions versus placebo of − 1.9 and − 2.6 for 70 mg and 140 mg doses, respectively). In the current analysis, the corresponding relative reductions in the subgroup of users of MSM at baseline were − 2.4 and − 3.3 days. The efficacy of erenumab on MSMD in the MSM user subgroup appears to be better compared with efficacy on non-MSMD in non-MSM only users, possibly because erenumab reduces more severe migraine days that would be subject to MSM treatment. It should also be noted that the placebo response for non-MSM only users was higher, perhaps owing to these patients being a more preventive treatment-naïve population as reported previously [17, 18]. Overall, numerical differences consistently show an increased benefit with the erenumab 140 mg dose compared with the 70 mg dose across all endpoints reported.

### Effect of erenumab on MSMD response

Achievement of a given threshold of MSMD response, defined as reduction in monthly MSMD of ≥ 50 %, ≥ 75 and 100 %, represents a novel outcome measure, and given the importance of reducing acute medication overuse for preventing disease progression and managing CM, may represent a useful and complementary approach to assessing efficacy. In both the EM and CM studies, erenumab 70 mg was associated with approximately 40 % of patients achieving ≥ 50 % reduction in monthly MSMD, and erenumab 140 mg was associated with approximately 50 % of patients achieving ≥ 50 % reduction in monthly MSMD. In general, erenumab was associated with a higher proportion of patients achieving ≥ 50 %, ≥ 75 and 100 % reduction in monthly MSMD in both EM and CM, although 100 % response represents a particularly stringent goal, especially in patients with CM.

### Placebo rates for the MSMD versus other endpoints

Interestingly, the placebo response for the MSMD endpoint used in these analyses is lower than that for change from baseline in non-MSMD as well as that observed for some other endpoints, including MMD. For example, the reduction in MMD measured in the placebo groups of the EM study [11] (mean MMD over Months 4, 5 and 6 where MMD is a monthly measure for EM) and over Month 3 of the CM study [12] were.

–1.8 and − 4.2 compared with reductions of − 0.2 and − 1.6 in monthly MSMD, respectively.

The low placebo response on the MSM endpoint has been a consistent observation in erenumab trials [11, 12, 19–22]. This observation can be interpreted in light of the evidence that expectation bias may be a driver of placebo response [23]. More severe migraine requiring patients to take an MSM as opposed to a non-MSM may be associated with less expectation bias and/or the subpopulation of patients using MSMs may generally have less expectation bias. In line with this, the proportion of patients who were preventive treatment-naïve in this study was approximately twice as high in the non-MSM only users versus the MSM users for both EM and CM, which may have contributed to the higher placebo response in this subgroup [17, 18].

### Effect and clinical implications of erenumab on acute medication use

A substantial use of acute medication at baseline was observed for patients enrolled in the EM and CM studies, and the demonstration of reduction of MSM and non-MSM with erenumab treatment reflects either reduced frequency of headache events and/or reduced severity of events. Not surprisingly, the use of acute medication was higher in patients with CM than in patients with EM, and this wider window of therapeutic opportunity is likely responsible for the greater reductions in acute medication use observed in patients with CM compared with the EM population.

For those with EM, reduction in acute medication use mitigates a key risk factor for the conversion from EM to CM and the development of MOH [24]. Conversely, and in line with the results presented here, erenumab has also been associated with reductions in medication overuse in the CM study [25], and also with conversion from CM to EM [16].

The effects of onabotulinumtoxinA, another migraine preventive therapy, on acute headache medication use have also been reported from registrational studies. In patients with CM and medication overuse, onabotulinumtoxinA lowered acute triptan use but not combination analgesic or simple analgesic use [26, 27]. In contrast, although not limited to CM and MOH, erenumab lowered all acute medication use and this difference may have clinical implications. This has recently been reflected in a number of real-world studies of adult patients with migraine [28–33]. In a retrospective longitudinal cohort study involving patients with ≥ 1 erenumab claim over a period of 12 months, acute medication was generally discontinued within 5–7 months as a result of erenumab initiation [28]. Additionally, retrospective analyses of patients identified from the Optum De-identified Electronic Health Record database in the US have shown a decrease in acute medication use over time [29] as well as significant decreases in the number of types of acute medication used, and the number of claims of each medication, over a period of 6 months following erenumab initiation [30]. Other analyses of patients with CM, including an Italian observational study, revealed that a monthly, six-dose course of erenumab (70 mg or 140 mg) resulted in a reduction in analgesic use and triptan use [31]. Likewise, data from three Australian headache centers showed that patients with CM, who experienced ≥ 3 previous migraine preventive treatment failures or intolerances, saw reductions of 4 days in mean monthly triptan use and 2.7 days in mean monthly codeine use after 6 months of erenumab treatment [32]. Initial real-world evidence from a German headache center has also demonstrated that migraine patients treated with erenumab over a period of 3 months experience a reduction in acute medication and frequency of use [33]. Although more investigations beyond retrospective analyses are needed, these real-world findings support the effectiveness of erenumab in reducing acute medication use, in line with the results presented here.

## Conclusions

These post-hoc analyses of the EM and CM clinical studies show significant reductions in monthly acute HMD, as well as numerical reductions in non-MSMD in non-MSM only users at baseline, following erenumab treatment. Additionally, reductions in MSMD versus placebo were maintained in the extension phases of both studies. In conclusion, erenumab treatment of patients with EM and CM is associated with a significant and sustained reduction in the use of acute headache medications, including MSM, which may have clinical implications.

## Data Availability

Qualified researchers may request data from Amgen clinical studies. Complete details are available at amgen.com/datasharing.

## Abbreviations

AMPP: American Migraine Prevalence and Prevention
ATP: Active treatment phase
CGRP: Calcitonin gene–related peptide
CI: Confidence interval
CM: Chronic migraine
CMH: Cochran-Mantel-Haenszel
DBTP: Double-blind treatment phase
EM: Episodic migraine
ICHD-3: International Classification of Headache Disorders 3rd edition
mAb: Monoclonal antibody
MAST: Migraine in America Symptoms and Treatment
MMD: Monthly migraine days
MOH: Medication overuse headache
MSM: Migraine-specific medication
MSMD: Migraine-specific medication treatment days
NS: Non-significant
NSAID: Non-steroidal anti-inflammatory drug
OLTP: Open-label treatment phase
OTC: Over-the-counter
QM: Once-monthly
SE: Standard error
